# The healthcare costs of heart failure during the last five years of life: A retrospective cohort study

**DOI:** 10.23889/ijpds.v1i1.339

**Published:** 2017-04-19

**Authors:** William Hollingworth, Mousumi Biswas, Rachel Maishman, Mark Dayer, Theresa McDonagh, Sarah Purdy, Barnaby Reeves, Chris Rogers, Rachael Williams, Maria Pufulete

**Affiliations:** 1 London School of Hygiene and Tropical Medicine; 2 Public Health England; 3 Department of Cardiology, Taunton and Somerset NHS Foundation Trust; 4 King's College Hospital; 5 Clinical Practice Research Datalink

## Objectives

Evidence on the economic impact of heart failure (HF) is vital in order to predict the cost-effectiveness of novel interventions. The objective was to estimate the healthcare costs of HF during the last five years of life. 

## Approach

Adults who died with HF in 2012/3 were identified through linked English Office of National Statistics mortality data and Clinical Practice Research Datalink (CPRD) primary care data. CPRD and linked Hospital Episode Statistics admissions data were used to estimate the cost of primary care prescriptions and primary care and hospital admission healthcare with 95% confidence intervals (CI). Generalized least squares regression was used to estimate the relationship between costs, HF diagnosis and patient characteristics. 

## Results

In the last 90 days of life of 1,555 identified patients, healthcare costs were £8,912 (95% CI £8,436-9,388)per patient, more than 90% of which were for inpatient or critical care. In the last 90 days, patients spent on average 17.8 days (95% CI 16.8-18.8) in hospital and had 8.8 (95% CI 8.4-9.1) primary care consultations. Most (59%) patients were in hospital on the day of death. Mean quarterly healthcare costs were significantly higher after diagnosis than preceding diagnosis (by £1,479, 95% CI £1,286-1,671). Younger patients and patients with higher comorbidity had higher costs. 

## Conclusion

Healthcare costs increased sharply at the end of life and were dominated by hospital care. There is potential to save money by implementation and evaluation of interventions known to reduce HF hospitalisations, particularly at the end of life.

